# Screening for New Delhi metallo-β-lactamase-1 in Enterobacteriaceae: Is there a role for the modified Hodge test?

**DOI:** 10.12669/pjms.316.8159

**Published:** 2015

**Authors:** Nor Zanariah Zainol Abidin, Anita Sulong, Hanafiah Alfizah, Chuan Hun Ding, Najihan Abdul Samat Muttaqillah, Md Mostafizur Rahman

**Affiliations:** 1 Nor Zanariah Zainol Abidin, Department of Medical Microbiology & Immunology, Universiti Kebangsaan Malaysia Medical Centre, Kuala Lumpur, Malaysia; 2 Anita Sulong, Department of Medical Microbiology & Immunology, Universiti Kebangsaan Malaysia Medical Centre, Kuala Lumpur, Malaysia; 3 Hanafiah Alfizah, Department of Medical Microbiology & Immunology, Universiti Kebangsaan Malaysia Medical Centre, Kuala Lumpur, Malaysia; 4 Chuan Hun Ding, Department of Medical Microbiology & Immunology, Universiti Kebangsaan Malaysia Medical Centre, Kuala Lumpur, Malaysia; 5 Najihan Abdul Samat Muttaqillah, Department of Medical Microbiology & Immunology, Universiti Kebangsaan Malaysia Medical Centre, Kuala Lumpur, Malaysia; 6 Md Mostafizur Rahman. Department of Medical Microbiology & Immunology, Universiti Kebangsaan Malaysia Medical Centre, Kuala Lumpur, Malaysia

**Keywords:** Enterobacteriaceae, metallo-β-lactamase-1, NDM-1, modified Hodge test, MHT

## Abstract

**Objective:**

The New Delhi metallo-β-lactamase-1 (NDM-1) enzyme is a plasmid-encoded enzyme that inactivates carbapenem antibiotics. This study aims to ascertain if the modified Hodge test (MHT) has a role in screening for NDM-1 in Enterobacteriaceae with reduced carbapenem susceptibility.

**Methods:**

Over a period of one year, all Enterobacteriaceae isolates from all clinical specimens with reduced susceptibility to at least one carbapenem were subjected to MHT and conventional polymerase chain reaction (PCR) detection of the NDM-1 gene.

**Results:**

A total of 13,098 Enterobacteriaceae isolates were screened and 63 (0.48%) had reduced susceptibility to at least one carbapenem. Out of the 63 isolates, 45 (71.4%) were MHT-positive. The NDM-1 gene was detected in 18 of the 63 isolates (28.6%). All 18 PCR-positive isolates were also MHT-positive. Thus, the sensitivity and specificity of the MHT in detecting NDM-1 in Enterobacteriaceae with reduced carbapenem susceptibility are 100% and 40%, respectively.

**Conclusion:**

The MHT is a useful test to screen for the presence of NDM-1 in Enterobacteriaceae with reduced carbapenem susceptibility. However, due to its rather low specificity, all MHT-positive isolates should be subjected to alternative tests (e.g. PCR) for confirmation, especially if other types of carbapenemases (e.g. KPC) are prevalent.

## INTRODUCTION

New Delhi metallo-β-lactamase-1 (NDM-1) is a carbapenemase enzyme that hydrolyzes all β-lactam antibiotics except aztreonam. It was first described in 2008 in a 59-year-old Swedish patient when he was hospitalized for a urinary tract infection in New Delhi, India.^1^ Many NDM-1-positive isolates also harbour other resistant genes and pose major therapeutic dilemmas. Few new antimicrobials are in the pipeline to be used against carbapenem-resistant Enterobacteriaceae. Being carried on plasmids, the NDM-1 gene is readily transferable to other bacteria.^2^ Therefore, it is important to screen for NDM-1 production in Enterobacteriaceae so that rapid infection control measures can be instituted.

There is a phenotypic confirmatory test for carbapenemase production in Enterobacteriaceae known as the modified Hodge test (MHT). This test is relatively cheap, rapid and easy to perform and has been reported to have a detection sensitivity exceeding 90% for KPC-type carbapenemases and 11% for metallo-β-lactamases.^3^ However, other investigators have reported a higher detection sensitivity of 50% in NDM-1 producers.^4^ Thus, the aim of our study was to ascertain if the MHT can be used as a rapid screening method for NDM-1 production in our local Enterobacteriaceae population. This is especially important in our setting where the prevalence of KPC-type carbapenemases is low.

## METHODS

### Study design and population

This cross sectional study was conducted in Universiti Kebangsaan Malaysia Medical Centre (UKMMC) over a period of one year. All confirmed Enterobacteriaceae isolates (i.e. Gram-negative rods which were oxidase-negative and produced acidic reactions in triple-sugar iron agar) from clinical specimens sent to the microbiology laboratory for routine culture and antibiotic susceptibility testing during the study period were screened. When a bacterial isolate with an identical antibiogram was isolated more than once from a given patient, only the first isolate was included in the study.

### Speciation of Enterobacteriaceae

Identification was achieved with API 20E strips (bioMerieux, France) in accordance to the Kit’s product insert.

### Screening for carbapenem resistance

Screening was carried out by disk diffusion according to the recommendations in the Clinical and Laboratory Standards Institute (CLSI) document M100-S24.^3^ The carbapenem antibiotic disks used for each isolate were ertapenem (10mg), imipenem (10mg), meropenem (10mg) and doripenem (10mg). The susceptibility results were also interpreted according to the same document.^3^ Isolates with reduced susceptibility (i.e. testing resistant or intermediate) to one or more carbapenems were subjected to both MHT and PCR detection of the NDM-1 gene.

### Modified Hodge Test

**Fig.1:**
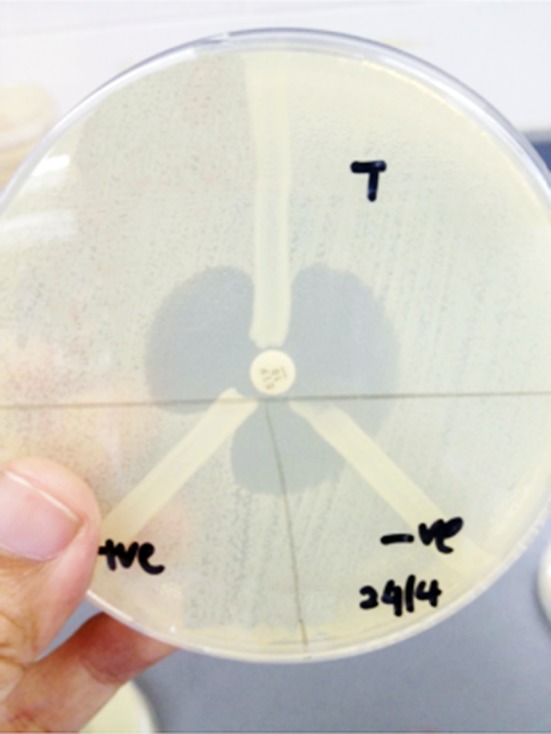
The MHT is positive when the indicator organism has enhanced growth at the intersection of the test (T) organism streak and the zone of inhibition. The positive control (+ve) and negative control (-ve) organism streaks are at 8 and 5 o’clock, respectively.

Prior to the MHT, a bacterial lawn of *Escherichia coli* ATCC® 25922 (the indicator organism) on Muller-Hinton agar was prepared. This lawn was essentially a 0.5 McFarland suspension of the indicator organism in Muller-Hinton broth that had been diluted further to 1:10. Once the *Escherichia coli* lawn was ready, an ertapenem (10 mg) disk was placed at the centre of the agar plate. With a sterile cotton swab, 3-5 colonies of the test organism were streaked in a singular motion and in a straight line starting from the edge of the ertapenem disk to the edge of the plate, with the width of the streak between 20 to 25 mm. Using another sterile cotton swab, 3-5 colonies of *Klebsiella pneumoniae* ATCC® BAA-1705 (the positive control organism) were streaked in a similar manner to the first but 120° away from it. Finally, a third streak of 3-5 colonies of *Klebsiella pneumoniae* ATCC® BAA-1706 (the negative control organism) was made 120° away from second streak. Each of the three organisms (one test organism and both control organisms) were grown overnight on a separate blood agar plate before they were streaked onto the same Muller-Hinton agar plate. The streaked plate was then incubated for 16-20 hours in ambient air at 35 ± 2°C. The presence of enhanced growth at the intersection of the streak and the zone of inhibition was interpreted as positive for carbapenemase production (Fig.1).

### PCR detection of the NDM-1 gene

Prior to performing PCR, bacterial DNA was extracted using Genomic DNA Mini Kit (Geneaid Biotech Ltd, Taiwan). For each isolate, overnight bacterial colonies were transferred from MacConkey agar into 3.0 mL of Trypticase-soy broth in a sterile container. The inoculum was incubated in 37°C for 16-20 hours prior to DNA extraction in accordance to the extraction kit’s protocol.

**Fig.2:**
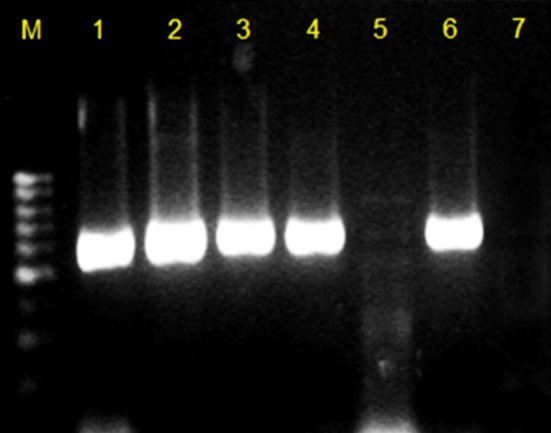
PCR products from the molecular detection of NDM-1 on agarose gel. Lane M is a 100-base-pair DNA molecular weight ladder, lane 1 is the positive control (621 base pairs) and lane 7 is the negative control. Each lane from 2 to 6 contains DNA from a different Enterobacteriaceae isolate. Samples in lanes 2, 3, 4 and 6 are positive for the NDM-1 gene.

The DNA was amplified using iCycler Thermo Cycler (Bio-Rad Laboratories, USA). The primers used are listed in Table-I and were described by Nordmann et al.^4^ The amplification was carried out under the following optimised thermal cycling conditions: 10 minutes at 95°C; 36 cycles of amplification consisting of 30 seconds at 95°C, 40 seconds at 61.4°C, 50 seconds at 72°C; and 5 minutes at 72°C for the final extension. The DNA fragments were separated by gel electrophoresis at 90 V for 45 minutes. Amplified products were visualized under UV light after staining with GelRed dye (Fig.2).

**Table-I:** Primer sequences used for detection of the NDM-1 gene.

Primer	Sequence (5’ – 3’)	Amplicon size
Forward	GGT TTG GCG ATC TGG TTT TC	621 base
Reverse	CGG AAT GGC TCA TCA CGA TC	pairs

## RESULTS

A total of 13,098 Enterobactericeae isolates were screened during the study period. Out of this, 63 (0.48%) non-repetitive Enterobacteriaceae isolates had reduced susceptibility to at least one carbapenem. MHT and conventional PCR detection of the NDM-1 gene were performed on all the 63 isolates, and the results are as shown in Table-II. There is a statistically significant association between MHT positivity and the presence of the NDM-1 gene. Out of the 63, a total of 45 isolates were MHT-positive (71.4%). However, although the NDM-1 gene was detected in only 18 of the 63 isolates (28.6%), all the PCR-positive isolates were also MHT-positive. Thus, using PCR as the gold standard, the MHT’s calculated sensitivity and specificity for detecting NDM-1 in Enterobacteriaceae with reduced susceptibility to carbapenems were 100% and 40%, respectively.

**Table-II:** MHT and NDM-1 gene PCR results for Enterobactericeae isolates with reduced carbapenem susceptibility.

	NDM-1 gene detected	NDM-1 gene not detected	p-value*
MHT-positive	18 (28.6%)	27 (42.8%)	0.0013
MHT-negative	0 (0%)	18 (28.6%)	

* derived from Fisher’s exact test.

## DISCUSSION

Carbapenemase-producing Enterobacteriaceae cause serious infections in debilitated and immunocompromised patients and are associated with prolonged hospital stays and high mortality rates of up to 70%.^5^ The most clinically important carbapenemases in Enterobacteriaceae are the Ambler class A enzymes of the KPC type, the class B metallo-β-lactamases (represented mainly by the NDM, VIM, and IMP types) and the class D carbapenemases of the OXA-48 type.^5^,^6^

Prior to 2015, the MHT, which is also known as the cloverleaf test, was the only phenotypic confirmatory test for carbapenemase production in Enterobacteriaceae described by the CLSI.^3^ The MHT is based on the inactivation of a carbapenem by a carbapenemase-producing strain so that a carbapenem-susceptible indicator strain is able to grow towards a carbapenem disk along the streak line of the test strain.^5^ The carbapenem-producing strain (or positive control organism) recommended by the CLSI has a KPC type carbapenemase.^3^

The MHT has limitations as it is not able to distinguish the different types of carbapenemases and is fraught with false positive and false negative results.^6^ Thus, in order to increase the sensitivity of the MHT in screening for NDM type carbapenemases, other investigators have suggested modifications to the CLSI protocol. Replacing Mueller-Hinton agar with MacConkey agar or the addition of ZnSO_4_ (100 g/ml) to the culture medium are among some of the suggested modifications.^5^,^6^

Contrary to previous reports that the MHT has a low sensitivity (ranging from 11 to 50%) in detecting NDM-1, we found a high sensitivity of 100% when the test was performed on Enterobacteriaceae with reduced susceptibility to at least one carbapenem. However, our specificity was much lower, at only 40%. Our excellent sensitivity result is attributed to the absence of false-negatives (i.e. MHT-negative but PCR-positive). We achieved these results without having to perform any modification to the MHT procedure described by the CLSI.

Our finding of high MHT sensitivity is more consistent with that from a neighbouring country (Thailand) which reported that more than half of the NDM-1 positive Enterobacteriaceae isolates were MHT-positive.^7^ Compared to KPC-producing strains, NDM-1 producers have been known to give less strongly positive MHT results.^8^ This phenomenon was also observed in our NDM-1-producing isolates (Fig.1). Thus, laboratories that frequently encounter KPC-producing isolates may falsely report the positive MHT results of NDM-1 producers as negative. No KPC-producing isolates were reported in our medical centre during the study period. Similarly, the Thai investigators did not detect any KPC-positive Enterobacteriaceae in their screening isolates.^7^

### Limitation of the study

The limitation of our study is the relatively small sample size and that we specifically evaluated the role of MHT in detecting NDM-1. A more sensitive molecular detection method should have been utilized to enhance the detection of NDM-1 in our 18 isolates which were both MHT-negative as well as conventional PCR-negative. We also acknowledge that since NDM-1 was first described in 2008, many other NDM variants have been reported, the latest being NDM-14 in 2015.^9^ While it is possible that the results of our study can be extrapolated to other NDM variants as well, formal studies on this should be undertaken.

## CONCLUSION

The MHT is a useful test to screen for NDM-1-producers among Enterobacteriaceae with reduced susceptibility to at least one carbapenem in centres with a low prevalence of other carbapenemases (e.g. KPC). The test may be performed as per the CLSI protocol without the need for special disks or agar media. However, due to the relatively low specificity, all MHT-positive isolates should ideally also be subjected to alternative testing methods (e.g. PCR) to confirm the presence of NDM-1.

## References

[1] Yong D, Toleman MA, Giske CG, Cho HS, Sundman K, Lee K (2009). Characterization of a New Metallo-b-Lactamase Gene, bla_NDM-1_ and a Novel Erythromycin Esterase Gene Carried on a Unique Genetic Structure in Klebsiella pneumoniae Sequence Type 14 from India. Antimicrob Agents Chemother.

[2] Kumarasamy KK, Toleman MA, Walsh TR, Bagaria J, Butt F, Balakrishnan R (2010). Emergence of a new antibiotic resistance mechanism in India, Pakistan, and the UK: a molecular, biological, and epidemiological study. Lancet Infect Dis.

[3] CLSI (2014). Performance standard for antimicrobial susceptibility testing;twenty-third informational supplement. CLSI document M100-S24.

[4] Nordmann P, Poirel L, Carrër A, Toleman MA, Walsh TR (2011). How to detect NDM-1 producers. J Clin Microbiol.

[5] Girlich D, Poirel L, Nordmann P (2012). Value of the modified Hodge test for detection of emerging carbapenemases in Enterobacteriaceae. J Clin Microbiol.

[6] Tzouvelekis LS, Markogiannakis A, Psichogiou M, Tassios PT, Daikos GL (2012). Carbapenemases in Klebsiella pneumoniae and other Enterobacteriaceae: an evolving crisis of global dimensions. Clin Microbiol Rev.

[7] Rimrang B, Chanawong A, Lulitanond A, Wilailuckana C, Charoensri N, Sribenjalux P (2012). Emergence of NDM-1- and IMP-14a-producing Enterobacteriaceae in Thailand. J Antimicrob Chemother.

[8] Mochon AB, Garner OB, Hindler JA, Krogstad P, Ward KW, Lewinski MA (2011). New Delhi metallo-β-lactamase (NDM-1)-producing Klebsiella pneumoniae: case report and laboratory detection strategies. J Clin Microbiol.

[9] Zou D, Huang Y, Zhao X, Liu W, Dong D, Li H (2015). A novel New Delhi metallo-β-lactamase variant, NDM-14, isolated in a Chinese Hospital possesses increased enzymatic activity against carbapenems. Antimicrob Agents Chemother.

